# Versatile and multivalent nanobodies efficiently neutralize SARS-CoV-2

**DOI:** 10.1126/science.abe4747

**Published:** 2020-11-05

**Authors:** Yufei Xiang, Sham Nambulli, Zhengyun Xiao, Heng Liu, Zhe Sang, W. Paul Duprex, Dina Schneidman-Duhovny, Cheng Zhang, Yi Shi

**Affiliations:** 1 Department of Cell Biology, University of Pittsburgh, Pittsburgh, PA, USA.; 2 Center for Vaccine Research, University of Pittsburgh, Pittsburgh, PA, USA.; 3 Department of Microbiology and Molecular Genetics, University of Pittsburgh, Pittsburgh, PA, USA.; 4 Department of Pharmacology and Chemical Biology, University of Pittsburgh, Pittsburgh, PA, USA.; 5 University of Pittsburgh–Carnegie Mellon University Program in Computational Biology, Pittsburgh, PA, USA.; 6 School of Computer Science and Engineering, Institute of Life Sciences, The Hebrew University of Jerusalem, Jerusalem, Israel.

## Abstract

Cost-effective, efficacious therapeutics are urgently needed to combat the COVID-19 pandemic. In this study, we used camelid immunization and proteomics to identify a large repertoire of highly potent neutralizing nanobodies (Nbs) to the severe acute respiratory syndrome coronavirus 2 (SARS-CoV-2) spike protein receptor binding domain (RBD). We discovered Nbs with picomolar to femtomolar affinities that inhibit viral infection at concentrations below the nanograms-per-milliliter level, and we determined a structure of one of the most potent Nbs in complex with the RBD. Structural proteomics and integrative modeling revealed multiple distinct and nonoverlapping epitopes and indicated an array of potential neutralization mechanisms. We bioengineered multivalent Nb constructs that achieved ultrahigh neutralization potency (half-maximal inhibitory concentration as low as 0.058 ng/ml) and may prevent mutational escape. These thermostable Nbs can be rapidly produced in bulk from microbes and resist lyophilization and aerosolization.

## Nanobodies that neutralize

Monoclonal antibodies that bind to the spike protein of severe acute respiratory syndrome coronavirus 2 (SARS-CoV-2) show therapeutic promise but must be produced in mammalian cells and need to be delivered intravenously. By contrast, single-domain antibodies called nanobodies can be produced in bacteria or yeast, and their stability may enable aerosol delivery. Two papers now report nanobodies that bind tightly to spike and efficiently neutralize SARS-CoV-2 in cells. Schoof *et al.* screened a yeast surface display of synthetic nanobodies and Xiang *et al.* screened anti-spike nanobodies produced by a llama. Both groups identified highly potent nanobodies that lock the spike protein in an inactive conformation. Multivalent constructs of selected nanobodies achieved even more potent neutralization.

*Science*, this issue p. 1473, p. 1479

## Teaser

Potent neutralizers of SARS-CoV-2 are identified by screening either synthetic or llama-produced nanobodies.

Globally a novel, highly transmissible coronavirus—severe acute respiratory syndrome coronavirus 2 (SARS-CoV-2) (*1*, *2*)—has infected more than 30 million people and has claimed almost 1 million lives, with the numbers still rising as of September 2020. Despite preventive measures, such as quarantines and lockdowns that help curb viral transmission, the virus rebounds after social restrictions are lifted. Safe and effective therapeutics and vaccines remain urgently needed.

Like other zoonotic coronaviruses, SARS-CoV-2 expresses a surface spike (S) glycoprotein, which consists of S1 and S2 subunits that form a homotrimeric viral spike to interact with host cells. The interaction is mediated by the S1 receptor binding domain (RBD), which binds the peptidase domain of human angiotensin-converting enzyme 2 (hACE2) as a host receptor (*3*). Structural studies have revealed different conformations of the spike (*4*, *5*). In the prefusion stage, the RBD switches between a closed conformation and an open conformation for hACE2 interaction. In the postfusion stage, the S2 undergoes a substantial conformational change to trigger host membrane fusion (*6*). Investigations of sera from COVID-19 convalescent individuals have led to the identification of potent neutralizing antibodies (NAbs) that primarily target the RBD but also non-RBD epitopes (*7*–*13*). High-quality NAbs may overcome the risks of Fc-associated antibody-dependent enhancement and are promising therapeutic candidates (*14*, *15*).

V_H_H antibodies, or nanobodies (Nbs), are minimal, monomeric antigen binding domains derived from camelid single-chain antibodies (*16*). Unlike immunoglobulin G (IgG) antibodies, Nbs are small (~15 kDa), highly soluble and stable, readily bioengineered into bi- or multivalent forms, and amenable to low-cost, efficient microbial production. Owing to their robust physicochemical properties, Nbs can be administered by inhalation, which makes them appealing therapeutic agents for treatment of respiratory viruses (*17*, *18*). Recently, several SARS-CoV-2 neutralizing Nbs have been identified through the screening of SARS-CoV or Middle East respiratory syndrome (MERS) cross-reacting Nbs or the use of synthetic Nb libraries for RBD binding. However, these synthetic Nbs generally neutralize the virus at or below microgram-per-milliliter concentrations (*12*, *19*–*22*) and thus are hundreds of times less potent than the most effective NAbs, likely due to monovalency and/or lack of affinity maturation (*23*, *24*). The development of highly potent anti–SARS-CoV-2 Nbs may provide a means for versatile, cost-effective prophylaxis, therapeutics, and point-of-care diagnosis.

To produce high-quality SARS-CoV-2 neutralizing Nbs, we immunized a llama with the recombinant RBD. Compared with the preimmunized serum sample, the postimmunized serum showed potent and specific serologic activities toward RBD binding with a titer of 1.75 × 10^6^ (fig. S1A). The serum efficiently neutralized the pseudotyped SARS-CoV-2 at a half-maximal neutralization titer (NT_50_) of ~310,000 (fig. S1B), orders of magnitude higher than that of the convalescent sera obtained from recovered COVID-19 patients (*7*, *8*). To further characterize these activities, we separated the single-chain V_H_H antibodies from the IgGs. We confirmed that the single-chain antibodies achieve specific, high-affinity binding to the RBD and possess subnanometer half-maximal inhibitory concentration (IC_50_ = 509 pM) against the pseudotyped virus (fig. S1C).

**Fig. 1:**
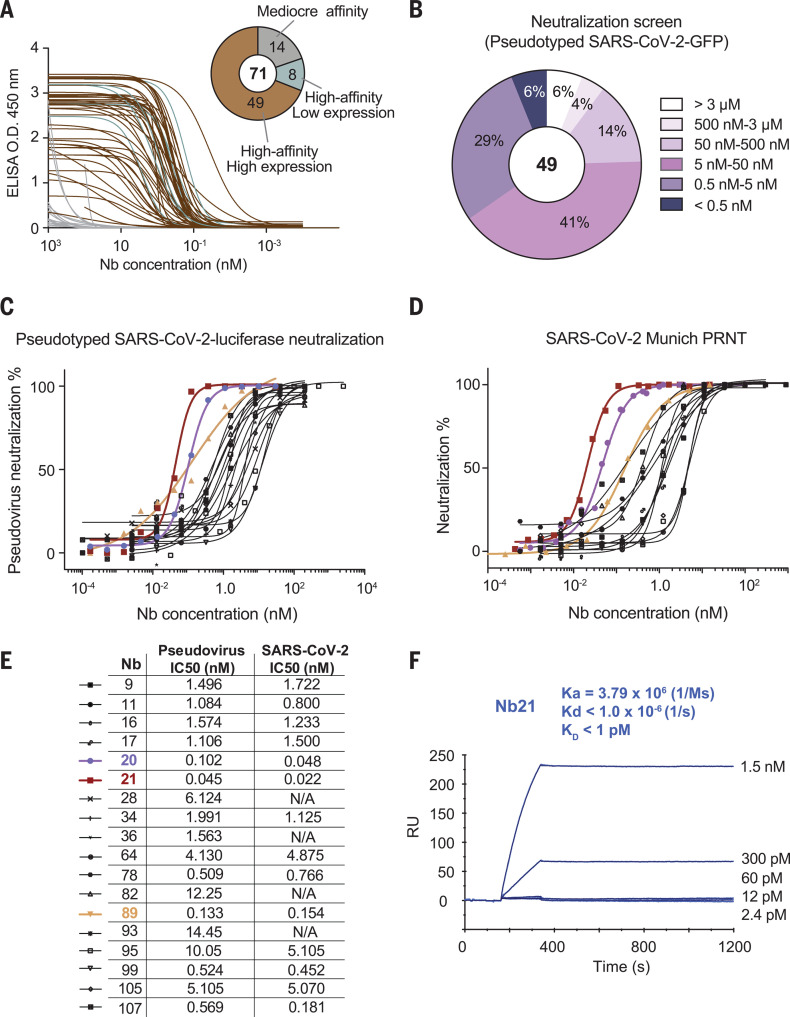
Production and characterizations of high-affinity RBD Nbs for SARS-CoV-2 neutralization. (**A**) Binding affinities of 71 Nbs toward the RBD, as determined by ELISA. The pie chart shows the number of Nbs according to affinity and solubility. O.D., optical density. (**B**) Screening of 49 high-affinity Nbs with high-expression level, as determined by SARS-CoV-2–GFP pseudovirus neutralization assay. *n* = 1 for Nbs with neutralization potency IC_50_ ≤ 50 nM; *n* = 2 for Nbs with neutralization potency IC_50_ > 50 nM (*n* indicates the number of replicates). (**C**) Neutralization potency of 18 highly potent Nbs was calculated on the basis of the pseudotyped SARS-CoV-2 neutralization assay (luciferase). Purple, red, and yellow lines denote Nbs 20, 21, and 89 with IC_50_ < 0.2 nM. Two different purifications of the pseudovirus were used. The average neutralization percentage was shown for each data point (*n* = 5 for Nbs 20 and 21; *n* = 2 for all other Nbs). (**D**) Neutralization potency of 14 neutralizing Nbs by SARS-CoV-2 plaque reduction neutralization test (PRNT). The average neutralization percentage was shown for each data point (*n* = 4 for Nbs 20, 21, and 89; *n* = 2 for other Nbs). (**E**) Summary table of pseudovirus and SARS-CoV-2 neutralization potencies of 18 Nbs. N/A, not tested. (**F**) SPR binding kinetics measurement for Nb21. Ka, acid dissociation constant; Kd, dissociation constant; K_D_, equilibrium dissociation constant; RU, relative units.

We identified thousands of high-affinity V_H_H Nbs from the RBD-immunized llama serum by using a robust proteomic strategy that we recently developed (*25*) (fig. S2A). This repertoire includes ~350 distinct CDR3s (CDRs, complementarity-determining regions). For *Escherichia coli* expression, we selected 109 highly diverse Nb sequences from the repertoire with distinct CDR3s to cover various biophysical, structural, and potentially different antiviral properties. Ninety-four Nbs were purified and tested for RBD binding by enzyme-linked immunosorbent assay (ELISA), from which we confirmed 71 RBD-specific binders (fig. S2, B and C, and tables S1 and S4). Of these RBD-specific binders, 49 Nbs presented high solubility and high affinity (ELISA IC_50_ below 30 nM; Fig. 1A) and were promising candidates for functional characterizations. We used a SARS-CoV-2–green fluorescent protein (GFP) pseudovirus neutralization assay to screen and characterize the antiviral activities of these high-affinity Nbs. Of the tested Nbs, 94% neutralize the pseudotype virus below 3 μM (Fig. 1B), and 90% neutralize below 500 nM. Only 20 to 40% of the high-affinity RBD-specific monoclonal antibodies identified from patient sera have been reported to possess comparable potency (*7*, *8*). More than three-quarters (76%) of the Nbs efficiently neutralized the pseudovirus below 50 nM, and 6% had neutralization activities below 0.5 nM. We selected the 18 most potent Nbs on the basis of the pseudovirus GFP reporter screen and measured their potency accurately using the pseudovirus-luciferase reporter assay. Finally, we used the plaque reduction neutralization test (PRNT) assay (*26*) to evaluate the potential of 14 Nbs to neutralize the SARS-CoV-2 Munich strain. All of the Nbs reached 100% neutralization and neutralized the virus in a dose-dependent manner. The IC_50_ values range from single-digit nanograms-per-milliliter amounts to below the nanograms-per-milliliter level. Of the most potent Nbs, three of them (89, 20, and 21) showed neutralization of 2.1 ng/ml (0.133 nM), 1.6 ng/ml (0.102 nM), and 0.7 ng/ml (0.045 nM), respectively, in the pseudovirus assay (Fig. 1C) and 0.154, 0.048, and 0.022 nM, respectively, in the SARS-CoV-2 assay (Fig. 1, D and E). Overall, there was an excellent correlation between the two neutralization assays (*R*^2^ = 0.92; fig. S3).

We measured the binding kinetics of Nbs 89, 20, and 21 by surface plasmon resonance (SPR) (fig. S4, A and B). Nbs 89 and 20 have affinities of 108 and 10.4 pM, respectively, and the most potent Nb21 did not show detectable dissociation from the RBD during 20 min of SPR analysis. The subpicomolar affinity of Nb21 potentially explains its unusual neutralization potency (Fig. 1F). From the *E. coli* periplasmic preparations, we determined the thermostability of Nbs 89, 20, and 21 to be 65.9°, 71.8°, and 72.8°C, respectively (fig. S4C). Finally, we tested the on-shelf stability of Nb21, which remained soluble after ~6 weeks of storage at room temperature after purification. No multimeric forms or aggregations were detected by size exclusion chromatography (SEC) (fig. S4D). Together, these results suggest that these neutralizing Nbs have valuable physicochemical properties for advanced therapeutic applications.

**Fig. 2:**
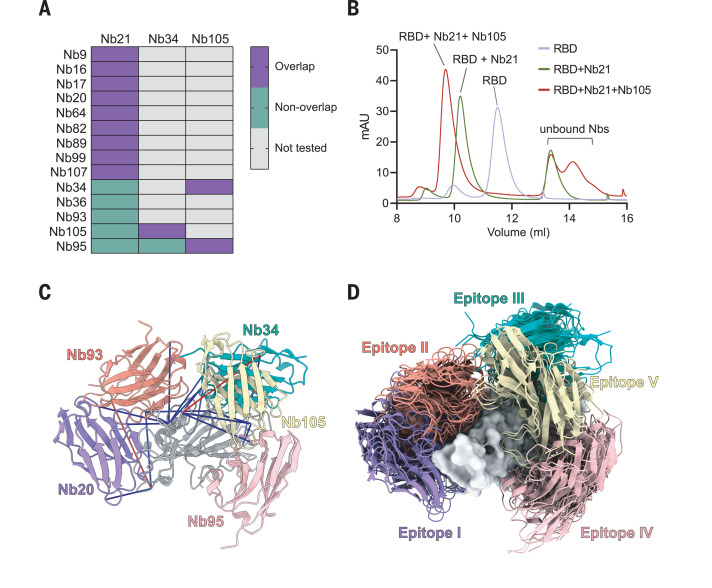
Nb epitope mapping by integrative structural proteomics. (**A**) Summary of Nb epitopes on the basis of SEC analysis. Purple, Nbs that bind the same RBD epitope; green, Nbs of different epitopes; gray, not tested. (**B**) Representation of SEC profiling of the RBD, RBD-Nb21 complex, and RBD-Nb21-Nb105 complex. The *y* axis represents ultraviolet 280 nm absorbance units (mAu). (**C**) Cartoon model showing the localization of five Nbs that bind different epitopes: Nb20 (purple), Nb34 (green), Nb93 (dark pink), Nb105 (yellow), and Nb95 (light pink) in complex with the RBD (gray). Blue and red lines represent DSS cross-links shorter or longer than 28 Å, respectively. (**D**) Top-10-scoring cross-linking–based models for each Nb (cartoons) on top of the RBD surface.

We employed an integrative approach by using SEC, cross-linking and mass spectrometry, and structural modeling for epitope mapping (*27*–*30*). First, we performed SEC experiments to distinguish between Nbs that share the same RBD epitope with Nb21 and those that bind to nonoverlapping epitopes. According to SEC profiles, Nbs 9, 16, 17, 20, 64, 82, 89, 99, and 107 competed with Nb21 for RBD binding (Fig. 2A and fig. S5), which indicates that their epitopes overlap substantially. By contrast, higher-mass species (from early elution volumes) corresponding to the trimeric complexes composed of Nb21, RBD, and one of the Nbs (34, 36, 93, 105, and 95) were evident (Fig. 2B and fig. S6, A to H). Moreover, Nb105 competed with Nbs 34 and 95, which did not compete for RBD interaction, suggesting the presence of two distinct and nonoverlapping epitopes. Second, we used disuccinimidyl suberate (DSS) to cross-link Nb-RBD complexes, and we used mass spectrometry to identify, on average, four intermolecular cross-links for Nbs 20, 93, 34, 95, and 105. The cross-links were used to map the RBD epitopes derived from the SEC data (materials and methods). Our cross-linking models identified five epitopes (I, II, III, IV, and V corresponding to Nbs 20, 93, 34, 95, and 105) (Fig. 2C). The models satisfied 90% of the cross-links with an average precision of 7.8 Å (Fig. 2D and table S2). Our analysis confirmed the presence of a dominant epitope I (e.g., epitopes of Nbs 20 and 21) overlapping with the hACE2 binding site. Epitope II also colocalized with the nonconserved hACE2 binding site. Both epitopes I and II Nbs can compete with hACE2 binding to the RBD at very low concentrations in vitro (fig. S7A). Epitopes III to V colocalized with conserved sites (fig. S7, B and C). Notably, epitope I Nbs had significantly shorter CDR3s (four amino acids shorter; *P* = 0.005) than other epitope binders (fig. S6I). Despite this, most of the selected Nbs potently inhibited the virus with an IC_50_ below 30 ng/ml (2 nM) (table S1).

**Fig. 3:**
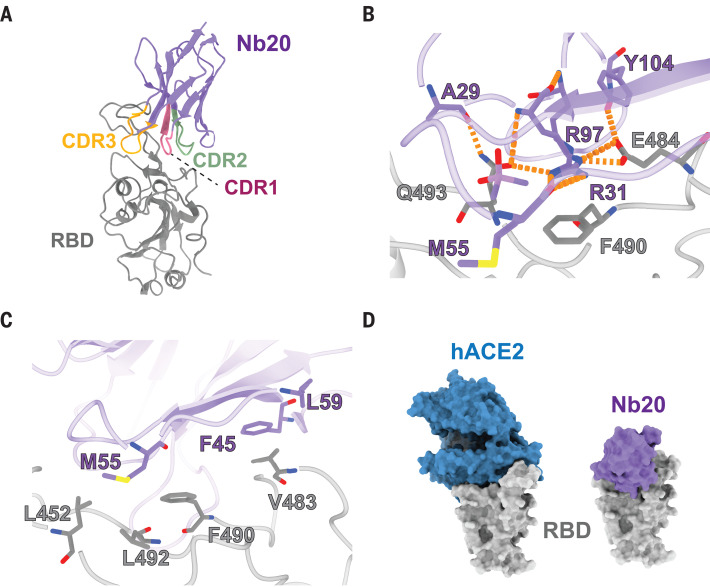
Crystal structure analysis of an ultrahigh-affinity Nb in complex with the RBD. (**A**) Cartoon presentation of Nb20 in complex with the RBD. CDR1, -2, and -3 are in red, green, and orange, respectively. (**B**) Zoomed-in view of an extensive polar interaction network that centers on R35 of Nb20. (**C**) Zoomed-in view of hydrophobic interactions. (**D**) Surface presentation of the Nb20-RBD and hACE2-RBD complex (PDB ID 6M0J). Single-letter abbreviations for amino acid residues are as follows: A, Ala; E, Glu; F, Phe; L, Leu; M, Met; Q, Gln; R, Arg; V, Val.

To explore the molecular mechanisms that underlie the potent neutralization activities of epitope I Nbs, we determined a crystal structure of the RBD-Nb20 complex at a resolution of 3.3 Å by molecular replacement (materials and methods, table S3, and fig. S13). Most of the residues in the RBD (Asn^334^ to Gly^526^) and in the entire Nb20, particularly those at the protein interaction interface, are well resolved. There are two nearly identical copies of RBD-Nb20 complexes in one asymmetric unit, with a root mean square deviation of 0.277 Å over 287 Cα atoms. In the structure, all three CDRs of Nb20 interact with the RBD by binding to its large extended external loop with two short β strands (Fig. 3A) (*31*). Glu^484^ of the RBD forms hydrogen bonding and ionic interactions with the side chains of Arg^31^ (CDR1) and Tyr^104^ (CDR3) of Nb20, whereas Gln^493^ of the RBD forms hydrogen bonds with the main-chain carbonyl of Ala^29^ (CDR1) and the side chain of Arg^97^ (CDR3) of Nb20. These interactions constitute a major polar interaction network at the RBD and Nb20 interface. Arg^31^ of Nb20 also engages in a cation π interaction with the side chain of Phe^490^ of the RBD (Fig. 3B). In addition, Met^55^ from the CDR2 of Nb20 packs against residues Leu^452^, Phe^490^, and Leu^492^ of the RBD to form hydrophobic interactions at the interface. Another small patch of hydrophobic interactions is formed among residues Val^483^ of the RBD and Phe^45^ and Leu^59^ from the framework β sheet of Nb20 (Fig. 3C).

The binding mode of Nb20 to the RBD is distinct from those of other reported neutralizing Nbs, which generally recognize similar epitopes in the RBD external loop region (*32*–*34*) (fig. S8). The extensive hydrophobic and polar interactions (Fig. 3, B and C) between the RBD and Nb20 stem from the notable shape complementarity (Fig. 3D) between the CDRs and the external RBD loop, leading to ultrahigh affinity (~10 pM). On the basis of our crystal structure, we further modeled the structure of the best neutralizer Nb21 with the RBD (materials and methods). Only four residues vary between Nbs 20 and 21 (fig. S9A), all of which are on CDRs. Two substitutions are at the RBD binding interface. Ser^52^ and Met^55^ in the CDR2 of Nb20 are replaced by two asparagine residues (Asn^52^ and Asn^55^) in Nb21. In our superimposed structure, Asn^52^ forms a new H bond with Asn^450^ of the RBD (fig. S9B). Although Asn^55^ does not engage in additional interactions with the RBD, it creates a salt bridge with the side chain of Arg^31^, which stabilizes the polar interaction network among Arg^31^ and Tyr^104^ of Nb21 and Gln^484^ of the RBD (fig. S9B). All of those likely contribute to a slower off-rate of Nb21 (Fig. 1F and fig. S4A) and stronger neutralization potency. Structural comparison of RBD-Nb20 or RBD-Nb21 and RBD-hACE2 [Protein Data Bank (PDB) ID 6LZG] (*31*) clearly showed that the interfaces for Nb20 or Nb21 and hACE2 partially overlap (Fig. 3D and fig. S9C). Notably, the CDR1 and CDR3 of Nb20 or Nb21 would clash with the first helix of hACE2, the primary binding site for the RBD (fig. S9D).

**Fig. 4:**
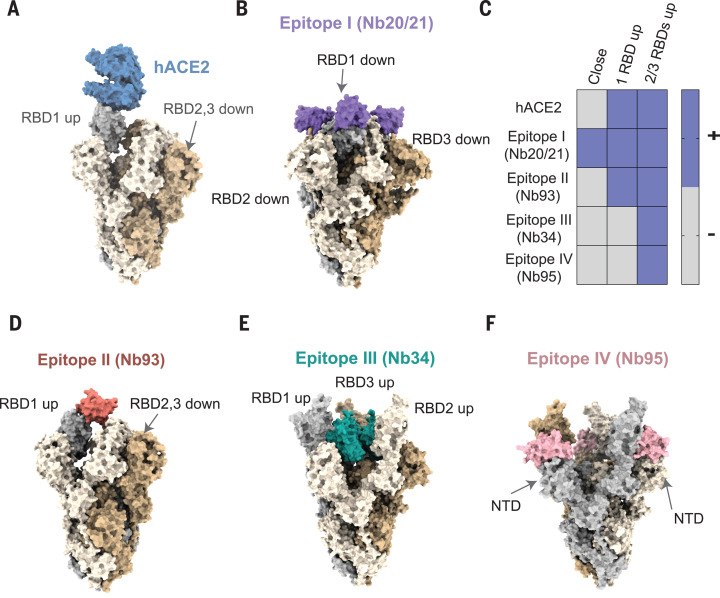
Potential mechanisms of SARS-CoV-2 neutralization by Nbs. (**A**) hACE2 (blue) binding to spike trimer conformation (wheat, beige, and gray colors) with one RBD in the “up” conformation (PDB IDs 6VSB and 6LZG). (**B**) Nb20 (epitope I, purple) partially overlaps with the hACE2 binding site and can bind the closed spike conformation with all RBDs “down” (PDB ID 6VXX). (**C**) Summary of spike conformations accessible (+) to the Nbs of different epitopes. (**D**) Nb93 (epitope II, dark pink) partially overlaps with the hACE2 binding site and can bind to spike conformations with at least one RBD up (PDB ID 6VSB). (**E** and **F**) Nb34 (epitope III, green) and Nb95 (epitope IV, light pink) do not overlap with the hACE2 binding site and bind to spike conformations with at least two open RBDs (PDB ID 6XCN).

To understand the antiviral efficacy of our Nbs, we superimposed RBD-Nb complexes on different spike conformations according to cryo–electron microscopy (cryo-EM) structures. We found that three copies of Nb20 or Nb21 can simultaneously bind all three RBDs in their “down” conformations (PDB ID 6VXX) (*4*) that correspond to the inactive spike (Fig. 4B). Our analysis indicates a potential mechanism by which Nbs 20 and 21 (epitope I) lock RBDs in their down conformation with ultrahigh affinity. Combined with the steric interference with hACE2 binding in the RBD open conformation (Fig. 4A), these mechanisms may explain the exceptional neutralization potencies of epitope I Nbs.

Other epitope binders do not fit into this inactive conformation without steric clashes and appear to use different neutralization strategies (Fig. 4C). For example, epitope II (Nb93) colocalizes with the hACE2 binding site and can bind the spike in the one RBD “up” conformation (Fig. 4D; PDB ID 6VSB) (*3*). This epitope may neutralize the virus by blocking the hACE2 binding site. Epitope III and IV Nbs can bind only when two or three RBDs are in their up conformations (PDB ID 6XCN) (*24*), in which the epitopes are exposed. In the all-RBDs-up conformation, three copies of Nbs can directly interact with the trimeric spike. Through RBD binding, epitope III (Nb34) can be accommodated on top of the trimer to lock the helices of S2 in the prefusion stage, possibly preventing their large conformational changes for membrane fusion (Fig. 4E). When superimposed onto the all-up conformation, epitope IV (Nb95) is proximal to the rigid N-terminal domain (NTD) of the trimer, presumably restricting the flexibility of the spike domains (Fig. 4F). Future high-resolution structural studies (e.g., by cryo-EM) of these Nbs in complex with the viral S protein will be needed to better understand the neutralization mechanisms.

Epitope mapping enabled us to bioengineer homo- and heterodimeric and homotrimeric Nbs. Homodimers and -trimers based on Nb20 or Nb21 were designed to increase the antiviral activities through avidity binding to the trimeric spike. Heterodimers pairing Nb21 with Nbs that bind a different epitope were designed to prevent viral escape. The homodimers and -trimers used flexible linker sequences of 25 (GS) or 31 (EK) amino acids (materials and methods). The heterodimers used flexible linkers of 12 amino acids.

**Fig. 5:**
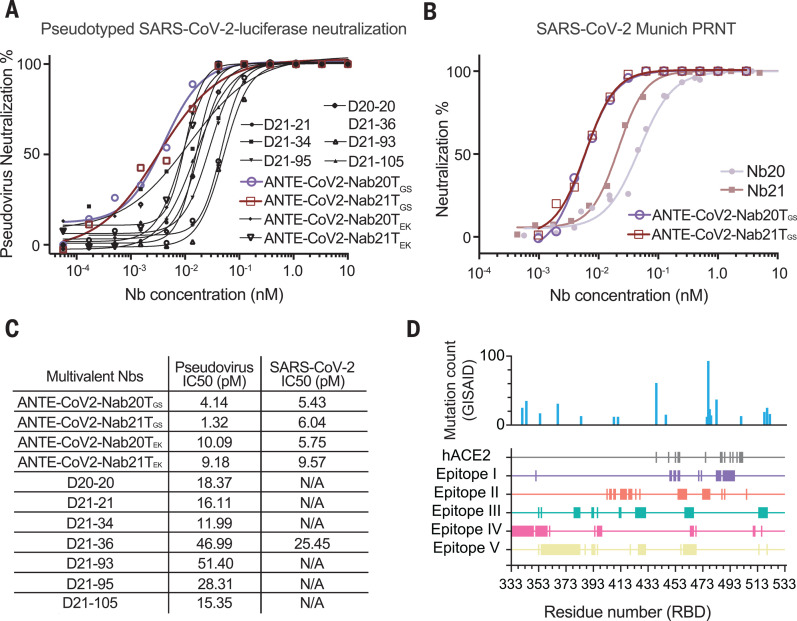
Development of multivalent Nb cocktails for highly efficient SARS-CoV-2 neutralization. (**A**) Pseudotyped SARS-CoV-2 neutralization assay of multivalent Nbs. The average neutralization percentage of each data point is shown (*n* = 2). ANTE-CoV2-Nab20T_GS/EK_: homotrimeric Nb20 with the GS or EK linker; ANTE-CoV2-Nab21T_GS/EK_: homotrimeric Nb21 with the GS or EK linker. (**B**) SARS-CoV-2 PRNT of monomeric and trimeric forms of Nbs 20 and 21. The average neutralization percentage of each data point is shown (*n* = 2 for the trimers; *n* = 4 for the monomers). (**C**) Summary table of the neutralization potency measurements of the multivalent Nbs. N/A, not tested. (**D**) Mapping mutations to localization of Nb epitopes on the RBD. The *x* axis corresponds to the RBD residue numbers (333 to 533). Rows in different colors represent different epitope residues. Epitope I: 351, 449 to 450, 452 to 453, 455 to 456, 470, 472, 483 to 486, and 488 to 496; epitope II: 403, 405 to 406, 408, 409, 413 to 417, 419 to 421, 424, 427, 455 to 461, 473 to 478, 487, 489, and 505; epitope III: 53, 355, 379 to 383, 392 to 393, 396, 412 to 413, 424 to 431, 460 to 466, and 514 to 520; epitope IV: 333 to 349, 351 to 359, 361, 394, 396 to 399, 464 to 466, 468, 510 to 511, and 516; epitope V: 353, 355 to 383, 387, 392 to 394, 396, 420, 426 to 431, 457, 459 to 468, 514, and 520.

Through a pseudovirus luciferase assay, we found up to ~30-fold improvement for the homotrimeric constructs of Nb21_3_ (IC_50_ = 1.3 pM) and Nb20_3_ (IC_50_ = 4.1 pM) compared with the respective monomeric form (Figs. 1, C and E, and 5, A and C). Similar results were obtained from the SARS-CoV-2 PRNT (Fig. 5, B and C, and fig. S11A). The improvements are likely greater than indicated by these values, as the measured values may reflect the assay’s lower detection limits. For the heterodimeric constructs (i.e., Nb21-Nb34), we observed up to a fourfold increase of potency. The multivalent constructs retained similar physicochemical properties to those of the monomeric Nbs (including high solubility, yield, and thermostability) and remained intact (nonproteolyzed) under the neutralization assay condition (fig. S10). They remained highly potent for pseudovirus neutralization after lyophilization and aerosolization (materials and methods and fig. S11, B to G), indicating the marked stability and potential flexibility of administration. Most of the RBD mutations observed in the Global Initiative on Sharing Avian Influenza Data (GISAID) (*35*) are very low in frequency (<0.0025), which may increase under Nb selection. Therefore, a cocktail consisting of ultrapotent, multivalent constructs that simultaneously bind a variety of epitopes with potentially different neutralization mechanisms will likely efficiently block virus mutational escape (Fig. 5E and fig. S12) (*9*, *36*–*38*).

In our study, in vivo antibody affinity maturation followed by advanced proteomics (*25*) enabled the rapid discovery of a diverse repertoire of high-affinity RBD Nbs, including an ultrapotent neutralizer with subpicomolar affinity, which is unusual for natural, single-domain antibody fragments. We demonstrated the simplicity and versatility of Nb bioengineering and the desirable physicochemical properties of the monomeric Nbs and their multivalent forms. To our knowledge, the multivalent constructs represent the most potent SARS-CoV-2 neutralizers to date. Flexible and efficient administration, such as inhalation, may further improve their antiviral efficacy while minimizing the dose, cost, and potential toxicity for clinical applications. The high sequence similarity between Nbs and human IgGs may restrain the immunogenicity (*39*). It is possible to fuse the antiviral Nbs with highly stable albumin-Nb constructs (*40*) to improve pharmacokinetics. These high-quality Nbs can also be applied as rapid and economic point-of-care diagnostics. We envision that the Nb technology described here will contribute to curbing the current pandemic and possibly a future event.

## Supplementary Materials

science.sciencemag.org/content/370/6523/1479/suppl/DC1 Materials and Methods Figs. S1 to S13 Tables S1 to S4 References (*41*–*60*) MDAR Reproducibility Checklist

## Appendix

View/request a protocol for this paper from *Bio-protocol*.

## References

[1] N. Zhu, D. Zhang, W. Wang, X. Li, B. Yang, J. Song, X. Zhao, B. Huang, W. Shi, R. Lu, P. Niu, F. Zhan, X. Ma, D. Wang, W. Xu, G. Wu, G. F. Gao, W. Tan; China Novel Coronavirus Investigating and Research Team, A Novel Coronavirus from Patients with Pneumonia in China, 2019. N. Engl. J. Med. 382, 727–733 (2020). 10.1056/NEJMoa2001017 31978945 PMC7092803

[2] P. Zhou, X.-L. Yang, X.-G. Wang, B. Hu, L. Zhang, W. Zhang, H.-R. Si, Y. Zhu, B. Li, C.-L. Huang, H.-D. Chen, J. Chen, Y. Luo, H. Guo, R.-D. Jiang, M.-Q. Liu, Y. Chen, X.-R. Shen, X. Wang, X.-S. Zheng, K. Zhao, Q.-J. Chen, F. Deng, L.-L. Liu, B. Yan, F.-X. Zhan, Y.-Y. Wang, G.-F. Xiao, Z.-L. Shi, A pneumonia outbreak associated with a new coronavirus of probable bat origin. Nature 579, 270–273 (2020). 10.1038/s41586-020-2012-7 32015507 PMC7095418

[3] D. Wrapp, N. Wang, K. S. Corbett, J. A. Goldsmith, C.-L. Hsieh, O. Abiona, B. S. Graham, J. S. McLellan, Cryo-EM structure of the 2019-nCoV spike in the prefusion conformation. Science 367, 1260–1263 (2020). 10.1126/science.abb2507 32075877 PMC7164637

[4] A. C. Walls, Y.-J. Park, M. A. Tortorici, A. Wall, A. T. McGuire, D. Veesler, Structure, Function, and Antigenicity of the SARS-CoV-2 Spike Glycoprotein. Cell 181, 281–292.e6 (2020). 10.1016/j.cell.2020.02.058 32155444 PMC7102599

[5] Y. Cai, J. Zhang, T. Xiao, H. Peng, S. M. Sterling, R. M. Walsh Jr.., S. Rawson, S. Rits-Volloch, B. Chen, Distinct conformational states of SARS-CoV-2 spike protein. Science 369, 1586–1592 (2020). 10.1126/science.abd4251 32694201 PMC7464562

[6] X. Fan, D. Cao, L. Kong, X. Zhang, Cryo-EM analysis of the post-fusion structure of the SARS-CoV spike glycoprotein. Nat. Commun. 11, 3618 (2020). 10.1038/s41467-020-17371-6 32681106 PMC7367865

[7] Y. Cao, B. Su, X. Guo, W. Sun, Y. Deng, L. Bao, Q. Zhu, X. Zhang, Y. Zheng, C. Geng, X. Chai, R. He, X. Li, Q. Lv, H. Zhu, W. Deng, Y. Xu, Y. Wang, L. Qiao, Y. Tan, L. Song, G. Wang, X. Du, N. Gao, J. Liu, J. Xiao, X. D. Su, Z. Du, Y. Feng, C. Qin, C. Qin, R. Jin, X. S. Xie, Potent Neutralizing Antibodies against SARS-CoV-2 Identified by High-Throughput Single-Cell Sequencing of Convalescent Patients’ B Cells. Cell 182, 73–84.e16 (2020). 10.1016/j.cell.2020.05.025 32425270 PMC7231725

[8] D. F. Robbiani, C. Gaebler, F. Muecksch, J. C. C. Lorenzi, Z. Wang, A. Cho, M. Agudelo, C. O. Barnes, A. Gazumyan, S. Finkin, T. Hägglöf, T. Y. Oliveira, C. Viant, A. Hurley, H.-H. Hoffmann, K. G. Millard, R. G. Kost, M. Cipolla, K. Gordon, F. Bianchini, S. T. Chen, V. Ramos, R. Patel, J. Dizon, I. Shimeliovich, P. Mendoza, H. Hartweger, L. Nogueira, M. Pack, J. Horowitz, F. Schmidt, Y. Weisblum, E. Michailidis, A. W. Ashbrook, E. Waltari, J. E. Pak, K. E. Huey-Tubman, N. Koranda, P. R. Hoffman, A. P. West Jr.., C. M. Rice, T. Hatziioannou, P. J. Bjorkman, P. D. Bieniasz, M. Caskey, M. C. Nussenzweig, Convergent antibody responses to SARS-CoV-2 in convalescent individuals. Nature 584, 437–442 (2020). 10.1038/s41586-020-2456-9 32555388 PMC7442695

[9] J. Hansen, A. Baum, K. E. Pascal, V. Russo, S. Giordano, E. Wloga, B. O. Fulton, Y. Yan, K. Koon, K. Patel, K. M. Chung, A. Hermann, E. Ullman, J. Cruz, A. Rafique, T. Huang, J. Fairhurst, C. Libertiny, M. Malbec, W. Y. Lee, R. Welsh, G. Farr, S. Pennington, D. Deshpande, J. Cheng, A. Watty, P. Bouffard, R. Babb, N. Levenkova, C. Chen, B. Zhang, A. Romero Hernandez, K. Saotome, Y. Zhou, M. Franklin, S. Sivapalasingam, D. C. Lye, S. Weston, J. Logue, R. Haupt, M. Frieman, G. Chen, W. Olson, A. J. Murphy, N. Stahl, G. D. Yancopoulos, C. A. Kyratsous, Studies in humanized mice and convalescent humans yield a SARS-CoV-2 antibody cocktail. Science 369, 1010–1014 (2020). 10.1126/science.abd0827 32540901 PMC7299284

[10] L. Liu, P. Wang, M. S. Nair, J. Yu, M. Rapp, Q. Wang, Y. Luo, J. F.-W. Chan, V. Sahi, A. Figueroa, X. V. Guo, G. Cerutti, J. Bimela, J. Gorman, T. Zhou, Z. Chen, K.-Y. Yuen, P. D. Kwong, J. G. Sodroski, M. T. Yin, Z. Sheng, Y. Huang, L. Shapiro, D. D. Ho, Potent neutralizing antibodies against multiple epitopes on SARS-CoV-2 spike. Nature 584, 450–456 (2020). 10.1038/s41586-020-2571-7 32698192

[11] P. J. M. Brouwer, T. G. Caniels, K. van der Straten, J. L. Snitselaar, Y. Aldon, S. Bangaru, J. L. Torres, N. M. A. Okba, M. Claireaux, G. Kerster, A. E. H. Bentlage, M. M. van Haaren, D. Guerra, J. A. Burger, E. E. Schermer, K. D. Verheul, N. van der Velde, A. van der Kooi, J. van Schooten, M. J. van Breemen, T. P. L. Bijl, K. Sliepen, A. Aartse, R. Derking, I. Bontjer, N. A. Kootstra, W. J. Wiersinga, G. Vidarsson, B. L. Haagmans, A. B. Ward, G. J. de Bree, R. W. Sanders, M. J. van Gils, Potent neutralizing antibodies from COVID-19 patients define multiple targets of vulnerability. Science 369, 643–650 (2020). 10.1126/science.abc5902 32540902 PMC7299281

[12] D. Wrapp, D. De Vlieger, K. S. Corbett, G. M. Torres, N. Wang, W. Van Breedam, K. Roose, L. van Schie, M. Hoffmann, S. Pöhlmann, B. S. Graham, N. Callewaert, B. Schepens, X. Saelens, J. S. McLellan; VIB-CMB COVID-19 Response Team, Structural Basis for Potent Neutralization of Betacoronaviruses by Single-Domain Camelid Antibodies. Cell 181, 1436–1441 (2020). 10.1016/j.cell.2020.05.047 32531248 PMC7289117

[13] A. Z. Wec, D. Wrapp, A. S. Herbert, D. P. Maurer, D. Haslwanter, M. Sakharkar, R. K. Jangra, M. E. Dieterle, A. Lilov, D. Huang, L. V. Tse, N. V. Johnson, C.-L. Hsieh, N. Wang, J. H. Nett, E. Champney, I. Burnina, M. Brown, S. Lin, M. Sinclair, C. Johnson, S. Pudi, R. Bortz 3rd, A. S. Wirchnianski, E. Laudermilch, C. Florez, J. M. Fels, C. M. O’Brien, B. S. Graham, D. Nemazee, D. R. Burton, R. S. Baric, J. E. Voss, K. Chandran, J. M. Dye, J. S. McLellan, L. M. Walker, Broad neutralization of SARS-related viruses by human monoclonal antibodies. Science 369, 731–736 (2020). 10.1126/science.abc7424 32540900 PMC7299279

[14] T. Zohar, G. Alter, Dissecting antibody-mediated protection against SARS-CoV-2. Nat. Rev. Immunol. 20, 392–394 (2020). 10.1038/s41577-020-0359-5 32514035 PMC7278217

[15] N. Eroshenko, T. Gill, M. K. Keaveney, G. M. Church, J. M. Trevejo, H. Rajaniemi, Implications of antibody-dependent enhancement of infection for SARS-CoV-2 countermeasures. Nat. Biotechnol. 38, 789–791 (2020). 10.1038/s41587-020-0577-1 32504046

[16] S. Muyldermans, Nanobodies: Natural single-domain antibodies. Annu. Rev. Biochem. 82, 775–797 (2013). 10.1146/annurev-biochem-063011-092449 23495938

[17] P. Vanlandschoot, C. Stortelers, E. Beirnaert, L. I. Ibañez, B. Schepens, E. Depla, X. Saelens, Nanobodies®: New ammunition to battle viruses. Antiviral Res. 92, 389–407 (2011). 10.1016/j.antiviral.2011.09.002 21939690

[18] L. Detalle, T. Stohr, C. Palomo, P. A. Piedra, B. E. Gilbert, V. Mas, A. Millar, U. F. Power, C. Stortelers, K. Allosery, J. A. Melero, E. Depla, Generation and Characterization of ALX-0171, a Potent Novel Therapeutic Nanobody for the Treatment of Respiratory Syncytial Virus Infection. Antimicrob. Agents Chemother. 60, 6–13 (2015). 10.1128/AAC.01802-15 26438495 PMC4704182

[19] R. Konwarh, Nanobodies: Prospects of Expanding the Gamut of Neutralizing Antibodies Against the Novel Coronavirus, SARS-CoV-2. Front. Immunol. 11, 1531 (2020). 10.3389/fimmu.2020.01531 32655584 PMC7324746

[20] T. F. Custódio, H. Das, D. J. Sheward, L. Hanke, S. Pazicky, J. Pieprzyk, M. Sorgenfrei, M. Schroer, A. Gruzinov, C. Jeffries, M. Graewert, D. Svergun, N. Dobrev, K. Remans, M. A. Seeger, G. M. McInerney, B. Murrell, B. M. Hällberg, C. Löw, Selection, biophysical and structural analysis of synthetic nanobodies that effectively neutralize SARS-CoV-2. bioRxiv 2020.06.23.165415 [Preprint]. 23 June 2020. 10.1101/2020.06.23.165415 PMC7642358 33149112

[21] C. Liu, Y. Yang, Y. Gao, C. Shen, B. Ju, C. Liu, X. Tang, J. Wei, X. Ma, W. Liu, S. Xu, Y. Liu, J. Yuan, J. Wu, Z. Liu, Z. Zhang, P. Wang, L. Liu, Viral Architecture of SARS-CoV-2 with Post-Fusion Spike Revealed by Cryo-EM. bioRxiv 2020.03.02.972927 [Preprint]. 5 March 2020. 10.1101/2020.03.02.972927

[22] J. Gai, L. Ma, G. Li, M. Zhu, P. Qiao, X. Li, H. Zhang, Y. Zhang, Y. Chen, W. Ji, H. Zhang, H. Cao, X. Li, R. Gong, Y. Wan, A potent neutralizing nanobody against SARS-CoV-2 with inhaled delivery potential. bioRxiv 2020.08.09.242867 [Preprint]. 14 August 2020. 10.1101/2020.08.09.242867 PMC8013425 33821254

[23] C. J. Bracken, S. A. Lim, P. Solomon, N. J. Rettko, D. P. Nguyen, B. S. Zha, K. Schaefer, J. R. Byrnes, J. Zhou, I. Lui, J. Liu, K. Pance, QCRG Structural Biology Consortium, X. X. Zhou, K. K. Leung, J. A. Wells, Bi-paratopic and multivalent human VH domains neutralize SARS-CoV-2 by targeting distinct epitopes within the ACE2 binding interface of Spike. bioRxiv 2020.08.08.242511 [Preprint]. 10 August 2020. 10.1101/2020.08.08.242511

[24] C. O. Barnes, A. P. West Jr.., K. E. Huey-Tubman, M. A. G. Hoffmann, N. G. Sharaf, P. R. Hoffman, N. Koranda, H. B. Gristick, C. Gaebler, F. Muecksch, J. C. C. Lorenzi, S. Finkin, T. Hägglöf, A. Hurley, K. G. Millard, Y. Weisblum, F. Schmidt, T. Hatziioannou, P. D. Bieniasz, M. Caskey, D. F. Robbiani, M. C. Nussenzweig, P. J. Bjorkman, Structures of Human Antibodies Bound to SARS-CoV-2 Spike Reveal Common Epitopes and Recurrent Features of Antibodies. Cell 182, 828–842.e16 (2020). 10.1016/j.cell.2020.06.025 32645326 PMC7311918

[25] Y. Xiang, Z. Sang, L. Bitton, J. Xu, Y. Liu, D. Schneidman-Duhovny, Y. Shi, Integrative proteomics reveals exceptional diversity and versatility of mammalian humoral immunity. bioRxiv 2020.08.21.261917 [Preprint]. 23 August 2020. 10.1101/2020.08.21.261917

[26] W. B. Klimstra, N. L. Tilston-Lunel, S. Nambulli, J. Boslett, C. M. McMillen, T. Gilliland, M. D. Dunn, C. Sun, S. E. Wheeler, A. Wells, A. L. Hartman, A. K. McElroy, D. S. Reed, L. J. Rennick, W. P. Duprex, SARS-CoV-2 growth, furin-cleavage-site adaptation and neutralization using serum from acutely infected hospitalized COVID-19 patients. J. Gen. Virol. 10.1099/jgv.0.001481 (2020). 10.1099/jgv.0.001481 32821033 PMC7879561

[27] M. P. Rout, A. Sali, Principles for Integrative Structural Biology Studies. Cell 177, 1384–1403 (2019). 10.1016/j.cell.2019.05.016 31150619 PMC6810593

[28] C. Yu, L. Huang, Cross-Linking Mass Spectrometry: An Emerging Technology for Interactomics and Structural Biology. Anal. Chem. 90, 144–165 (2018). 10.1021/acs.analchem.7b04431 29160693 PMC6022837

[29] A. Leitner, M. Faini, F. Stengel, R. Aebersold, Crosslinking and Mass Spectrometry: An Integrated Technology to Understand the Structure and Function of Molecular Machines. Trends Biochem. Sci. 41, 20–32 (2016). 10.1016/j.tibs.2015.10.008 26654279

[30] B. T. Chait, M. Cadene, P. D. Olinares, M. P. Rout, Y. Shi, Revealing Higher Order Protein Structure Using Mass Spectrometry. J. Am. Soc. Mass Spectrom. 27, 952–965 (2016). 10.1007/s13361-016-1385-1 27080007 PMC5125627

[31] Q. Wang, Y. Zhang, L. Wu, S. Niu, C. Song, Z. Zhang, G. Lu, C. Qiao, Y. Hu, K.-Y. Yuen, Q. Wang, H. Zhou, J. Yan, J. Qi, Structural and Functional Basis of SARS-CoV-2 Entry by Using Human ACE2. Cell 181, 894–904.e9 (2020). 10.1016/j.cell.2020.03.045 32275855 PMC7144619

[32] T. Li, H. Cai, H. Yao, B. Zhou, N. Zhang, Y. Gong, Y. Zhao, Q. Shen, W. Qin, C. A. J. Hutter, Y. Lai, S.-M. Kuo, J. Bao, J. Lan, M. A. Seeger, G. Wong, Y. Bi, D. Lavillette, D. Li, A potent synthetic nanobody targets RBD and protects mice from SARS-CoV-2 infection. bioRxiv 2020.06.09.143438 [Preprint]. 24 September 2020. 10.1101/2020.06.09.143438

[33] J. D. Walter, C. A. J. Hutter, I. Zimmermann, M. Wyss, P. Egloff, M. Sorgenfrei, L. M. Hürlimann, I. Gonda, G. Meier, S. Remm, S. Thavarasah, P. Plattet, M. A. Seeger, Sybodies targeting the SARS-CoV-2 receptor-binding domain. bioRxiv 2020.04.16.045419 [Preprint]. 16 May 2020. 10.1101/2020.04.16.045419

[34] J. Huo, A. Le Bas, R. R. Ruza, H. M. E. Duyvesteyn, H. Mikolajek, T. Malinauskas, T. K. Tan, P. Rijal, M. Dumoux, P. N. Ward, J. Ren, D. Zhou, P. J. Harrison, M. Weckener, D. K. Clare, V. K. Vogirala, J. Radecke, L. Moynié, Y. Zhao, J. Gilbert-Jaramillo, M. L. Knight, J. A. Tree, K. R. Buttigieg, N. Coombes, M. J. Elmore, M. W. Carroll, L. Carrique, P. N. M. Shah, W. James, A. R. Townsend, D. I. Stuart, R. J. Owens, J. H. Naismith, Neutralizing nanobodies bind SARS-CoV-2 spike RBD and block interaction with ACE2. Nat. Struct. Mol. Biol. 27, 846–854 (2020). 10.1038/s41594-020-0469-6 32661423

[35] Y. Shu, J. McCauley, GISAID: Global initiative on sharing all influenza data - from vision to reality. Euro Surveill. 22, 30494 (2017). 10.2807/1560-7917.ES.2017.22.13.30494 28382917 PMC5388101

[36] A. Baum, B. O. Fulton, E. Wloga, R. Copin, K. E. Pascal, V. Russo, S. Giordano, K. Lanza, N. Negron, M. Ni, Y. Wei, G. S. Atwal, A. J. Murphy, N. Stahl, G. D. Yancopoulos, C. A. Kyratsous, Antibody cocktail to SARS-CoV-2 spike protein prevents rapid mutational escape seen with individual antibodies. Science 369, 1014–1018 (2020). 10.1126/science.abd0831 32540904 PMC7299283

[37] Y. Bar-On, H. Gruell, T. Schoofs, J. A. Pai, L. Nogueira, A. L. Butler, K. Millard, C. Lehmann, I. Suárez, T. Y. Oliveira, T. Karagounis, Y. Z. Cohen, C. Wyen, S. Scholten, L. Handl, S. Belblidia, J. P. Dizon, J. J. Vehreschild, M. Witmer-Pack, I. Shimeliovich, K. Jain, K. Fiddike, K. E. Seaton, N. L. Yates, J. Horowitz, R. M. Gulick, N. Pfeifer, G. D. Tomaras, M. S. Seaman, G. Fätkenheuer, M. Caskey, F. Klein, M. C. Nussenzweig, Safety and antiviral activity of combination HIV-1 broadly neutralizing antibodies in viremic individuals. Nat. Med. 24, 1701–1707 (2018). 10.1038/s41591-018-0186-4 30258217 PMC6221973

[38] M. Marovich, J. R. Mascola, M. S. Cohen, Monoclonal Antibodies for Prevention and Treatment of COVID-19. JAMA 324, 131–132 (2020). 10.1001/jama.2020.10245 32539093

[39] I. Jovčevska, S. Muyldermans, The Therapeutic Potential of Nanobodies. BioDrugs 34, 11–26 (2020). 10.1007/s40259-019-00392-z 31686399 PMC6985073

[40] Z. Shen, Y. Xiang, S. Vegara, A. Chen, Z. Xiao, U. Santiago, C. Jin, Z. Sang, J. Luo, K. Chen, D. Schneidman-Duhovny, C. Camacho, G. Calero, B. Hu, Y. Shi, A robust and versatile nanobody platform for drug delivery. bioRxiv 2020.08.19.257725 [Preprint]. 20 August 2020. 10.1101/2020.08.19.257725

[41] P. C. Fridy, Y. Li, S. Keegan, M. K. Thompson, I. Nudelman, J. F. Scheid, M. Oeffinger, M. C. Nussenzweig, D. Fenyö, B. T. Chait, M. P. Rout, A robust pipeline for rapid production of versatile nanobody repertoires. Nat. Methods 11, 1253–1260 (2014). 10.1038/nmeth.3170 25362362 PMC4272012

[42] J. Dunbar, C. M. Deane, ANARCI: Antigen receptor numbering and receptor classification. Bioinformatics 32, 298–300 (2016). 26424857 10.1093/bioinformatics/btv552 PMC4708101

[43] S. Kumar, G. Stecher, M. Li, C. Knyaz, K. Tamura, MEGA X: Molecular Evolutionary Genetics Analysis across Computing Platforms. Mol. Biol. Evol. 35, 1547–1549 (2018). 10.1093/molbev/msy096 29722887 PMC5967553

[44] A. Tareen, J. B. Kinney, Logomaker: Beautiful sequence logos in Python. Bioinformatics 36, 2272–2274 (2020). 10.1093/bioinformatics/btz921 31821414 PMC7141850

[45] Y. Shi, J. Fernandez-Martinez, E. Tjioe, R. Pellarin, S. J. Kim, R. Williams, D. Schneidman-Duhovny, A. Sali, M. P. Rout, B. T. Chait, Structural characterization by cross-linking reveals the detailed architecture of a coatomer-related heptameric module from the nuclear pore complex. Mol. Cell. Proteomics 13, 2927–2943 (2014). 10.1074/mcp.M114.041673 25161197 PMC4223482

[46] Y. Shi, R. Pellarin, P. C. Fridy, J. Fernandez-Martinez, M. K. Thompson, Y. Li, Q. J. Wang, A. Sali, M. P. Rout, B. T. Chait, A strategy for dissecting the architectures of native macromolecular assemblies. Nat. Methods 12, 1135–1138 (2015). 10.1038/nmeth.3617 26436480 PMC4803312

[47] Y. Xiang, Z. Shen, Y. Shi, Chemical Cross-Linking and Mass Spectrometric Analysis of the Endogenous Yeast Exosome Complexes. Methods Mol. Biol. 2062, 383–400 (2020). 10.1007/978-1-4939-9822-7_18 31768986

[48] S. J. Kim, J. Fernandez-Martinez, I. Nudelman, Y. Shi, W. Zhang, B. Raveh, T. Herricks, B. D. Slaughter, J. A. Hogan, P. Upla, I. E. Chemmama, R. Pellarin, I. Echeverria, M. Shivaraju, A. S. Chaudhury, J. Wang, R. Williams, J. R. Unruh, C. H. Greenberg, E. Y. Jacobs, Z. Yu, M. J. de la Cruz, R. Mironska, D. L. Stokes, J. D. Aitchison, M. F. Jarrold, J. L. Gerton, S. J. Ludtke, C. W. Akey, B. T. Chait, A. Sali, M. P. Rout, Integrative structure and functional anatomy of a nuclear pore complex. Nature 555, 475–482 (2018). 10.1038/nature26003 29539637 PMC6022767

[49] A. Šali, T. L. Blundell, Comparative protein modelling by satisfaction of spatial restraints. J. Mol. Biol. 234, 779–815 (1993). 10.1006/jmbi.1993.1626 8254673

[50] A. Fiser, A. Sali, ModLoop: Automated modeling of loops in protein structures. Bioinformatics 19, 2500–2501 (2003). 10.1093/bioinformatics/btg362 14668246

[51] D. Schneidman-Duhovny, A. Rossi, A. Avila-Sakar, S. J. Kim, J. Velázquez-Muriel, P. Strop, H. Liang, K. A. Krukenberg, M. Liao, H. M. Kim, S. Sobhanifar, V. Dötsch, A. Rajpal, J. Pons, D. A. Agard, Y. Cheng, A. Sali, A method for integrative structure determination of protein-protein complexes. Bioinformatics 28, 3282–3289 (2012). 10.1093/bioinformatics/bts628 23093611 PMC3519461

[52] D. Schneidman-Duhovny, H. J. Wolfson, Modeling of Multimolecular Complexes. Methods Mol. Biol. 2112, 163–174 (2020). 10.1007/978-1-0716-0270-6_12 32006285

[53] G. Q. Dong, H. Fan, D. Schneidman-Duhovny, B. Webb, A. Sali, Optimized atomic statistical potentials: Assessment of protein interfaces and loops. Bioinformatics 29, 3158–3166 (2013). 10.1093/bioinformatics/btt560 24078704 PMC3842762

[54] Z. Otwinowski, W. Minor, Processing of X-ray diffraction data collected in oscillation mode. Methods Enzymol. 276, 307–326 (1997). 10.1016/S0076-6879(97)76066-X 27754618

[55] A. J. McCoy, R. W. Grosse-Kunstleve, P. D. Adams, M. D. Winn, L. C. Storoni, R. J. Read, Phaser crystallographic software. J. Appl. Crystallogr. 40, 658–674 (2007). 10.1107/S0021889807021206 19461840 PMC2483472

[56] P. D. Adams, P. V. Afonine, G. Bunkóczi, V. B. Chen, I. W. Davis, N. Echols, J. J. Headd, L.-W. Hung, G. J. Kapral, R. W. Grosse-Kunstleve, A. J. McCoy, N. W. Moriarty, R. Oeffner, R. J. Read, D. C. Richardson, J. S. Richardson, T. C. Terwilliger, P. H. Zwart, PHENIX: A comprehensive Python-based system for macromolecular structure solution. Acta Crystallogr. D 66, 213–221 (2010). 10.1107/S0907444909052925 20124702 PMC2815670

[57] P. Emsley, K. Cowtan, Coot: Model-building tools for molecular graphics. Acta Crystallogr. D 60, 2126–2132 (2004). 10.1107/S0907444904019158 15572765

[58] C. J. Williams, J. J. Headd, N. W. Moriarty, M. G. Prisant, L. L. Videau, L. N. Deis, V. Verma, D. A. Keedy, B. J. Hintze, V. B. Chen, S. Jain, S. M. Lewis, W. B. Arendall 3rd, J. Snoeyink, P. D. Adams, S. C. Lovell, J. S. Richardson, D. C. Richardson, MolProbity: More and better reference data for improved all-atom structure validation. Protein Sci. 27, 293–315 (2018). 10.1002/pro.3330 29067766 PMC5734394

[59] T. D. Goddard, C. C. Huang, E. C. Meng, E. F. Pettersen, G. S. Couch, J. H. Morris, T. E. Ferrin, UCSF ChimeraX: Meeting modern challenges in visualization and analysis. Protein Sci. 27, 14–25 (2018). 10.1002/pro.3235 28710774 PMC5734306

[60] F. H. Niesen, H. Berglund, M. Vedadi, The use of differential scanning fluorimetry to detect ligand interactions that promote protein stability. Nat. Protoc. 2, 2212–2221 (2007). 10.1038/nprot.2007.321 17853878

